# Age-Related Concentric Remodeling and Sex-Dependent Dimensional Variation in Left Ventricular Geometry: A Cardiac Magnetic Resonance Study

**DOI:** 10.3390/tomography12060090

**Published:** 2026-06-22

**Authors:** Davut Unsal Capkan, Mehmet Kaplan

**Affiliations:** 1 Department of Radiology, Medical Point Hospital, 27060 Gaziantep, Turkey; 2 Department of Cardiology, Medical Point Hospital, 27060 Gaziantep, Turkey; mehmet.kaplan@mphg.com.tr

**Keywords:** cardiac magnetic resonance, concentric remodeling, left ventricular geometry, wall thickness, aging, sex differences, end-diastolic diameter

## Abstract

Background/Objectives: Left ventricular (LV) geometry reflects structural adaptation to aging and biological sex. While cardiac magnetic resonance (CMR) provides precise morphologic assessment, most prior studies have focused on volumetric and mass-based parameters rather than routinely reported linear indices. This study aimed to evaluate the influence of age and sex on LV geometry using wall thickness, LV end-diastolic diameter (LVEDD), and proportional indices derived from standard CMR reports. Methods: In this retrospective cross-sectional study, 95 adult patients who underwent clinically indicated CMR were included. LV wall thickness, LVEDD, relative wall thickness (RWT), and wall thickness-to- LVEDD ratio (WT/LVEDD) were recorded. Participants were stratified by sex and age groups (18–40, 41–60, >60 years). Group comparisons, correlation analysis, multivariable linear regression, logistic regression, and Age × Sex interaction testing were performed to evaluate independent associated parameters of LV morphology and concentric remodeling. Results: The mean age was 34.94 ± 16.00 years; 60.0% were male. Males had significantly larger LVED (43.12 ± 6.83 mm vs. 39.76 ± 6.11 mm, *p* = 0.014) and greater wall thickness measurements (*p* < 0.05 for septal and posterior wall thickness). Age showed a significant positive correlation with mean LV wall thickness (r = 0.275, *p* = 0.007) and WT/LVEDD ratio (r = 0.241, *p* = 0.019), but not with LVEDD (*p* = 0.414). In multivariable analysis, male sex was independently associated with larger LVED (B = 3.345, *p* = 0.017), whereas age was independently associated with WT/LVEDD ratio (B = 0.0018, *p* = 0.019). Logistic regression demonstrated that age independently increased the odds of concentric remodeling (OR = 1.041 per year, 95% CI: 1.011–1.072, *p* = 0.006). No significant Age × Sex interaction was observed. Conclusions: Advancing age was independently associated with proportional LV geometric remodeling, whereas male sex primarily influenced absolute ventricular dimensions. Routine CMR report-derived linear measurements were sufficient to detect these distinct structural patterns. These findings highlighted the feasibility of using standardized morphologic indices in daily clinical practice to identify early age-related concentric remodeling.

## Simple Summary

The structure of the left ventricle changes over time due to aging and biological differences between men and women. While advanced cardiac imaging techniques provide detailed measurements, most clinical reports include simpler parameters such as wall thickness and chamber size. In this study, we analyzed routine cardiac magnetic resonance (CMR) data to understand how age and sex influence heart structure. We found that men tend to have larger heart chambers, whereas aging is associated with changes in the relative thickness of the heart wall, reflecting early structural adaptation. Importantly, these differences could be detected using standard measurements available in everyday clinical practice. Our findings suggest that simple CMR-derived parameters may help identify early changes in heart structure related to aging, potentially improving clinical assessment without the need for complex imaging analyses.

## 1. Introduction

Left ventricular (LV) geometry reflects the cumulative structural adaptation of the myocardium to physiological aging, hormonal influences, and hemodynamic loading conditions [1]. Subtle alterations in wall thickness and chamber dimensions often precede overt functional impairment and constitute early indicators of cardiac remodeling [2]. The characterization of these geometric patterns has therefore gained increasing clinical relevance, particularly in the context of risk stratification and subclinical cardiovascular disease detection [3].

Cardiac magnetic resonance (CMR) imaging is widely regarded as the gold standard for the assessment of cardiac morphology and structure due to its superior spatial resolution, high tissue contrast, and excellent reproducibility [4,5,6,7]. Unlike echocardiography, CMR allows precise quantification of myocardial wall thickness, LV end-diastolic diameter (LVEDD), and ventricular mass without geometric assumptions [8,9,10]. Standardized CMR protocols, as recommended by international societies, enable consistent and reliable morphological evaluation across diverse populations [11]. Furthermore, population-based imaging studies have highlighted the value of CMR in defining normative ranges for cardiac dimensions and functional parameters [12,13,14].

Aging is associated with progressive myocardial remodeling characterized by increased wall thickness, reduced chamber compliance, and alterations in ventricular geometry. These changes are thought to result from cumulative exposure to hemodynamic stress, vascular stiffening, and myocardial fibrosis [15,16,17]. Sex also plays a modifying role in cardiac structure. Men generally demonstrate larger LV dimensions and higher end-diastolic diameters, whereas women tend to exhibit relatively thicker walls in relation to cavity size, predisposing to concentric remodeling patterns [18,19]. Several large-scale CMR reference studies have confirmed age- and sex-specific variations in LV morphology [20,21,22].

Despite the availability of normative CMR reference values, studies directly investigating the combined influence of age and sex on LV geometric patterns using routinely reported morphological parameters remain limited. Most prior investigations have focused primarily on volumetric and functional indices rather than on simple structural metrics such as wall thickness and end-diastolic diameter derived from standard CMR reports [9,23,24,25,26]. However, these routinely measured parameters may carry substantial pathophysiological information regarding early remodeling processes.

Left ventricular geometry is commonly classified into four principal patterns—normal geometry, concentric remodeling, concentric hypertrophy, and eccentric hypertrophy—based on wall thickness and chamber dimensions [27,28]. The ratio between wall thickness and LV end-diastolic diameter (WT/LVEDD) provides a practical and reproducible index for geometric categorization [29,30]. Concentric remodeling, characterized by increased wall thickness relative to chamber size, has been associated with aging and pressure overload states, whereas eccentric hypertrophy is more often linked to volume overload conditions [31,32].

Understanding how age and sex influence these geometric patterns in real-world CMR data is clinically relevant for several reasons. First, distinguishing physiological aging-related remodeling from pathological hypertrophic processes requires robust reference data. Second, early geometric alterations may precede measurable declines in systolic function, offering a potential window for preventive intervention. Third, defining population-specific structural characteristics may improve the interpretability of routine CMR reports in daily clinical practice.

This study aimed to determine how age and sex influence left ventricular geometry defined by wall thickness and end-diastolic diameter measurements derived from routine cardiac magnetic resonance reports. Specifically, we sought to quantify age-related structural trends, compare sex-based differences in geometric parameters, and classify LV remodeling patterns using the wall thickness-to-diameter ratio. By focusing on standardized morphological indices obtained in daily clinical practice, the study aimed to clarify whether routine CMR measurements can reliably reflect early structural remodeling and contribute to the establishment of age- and sex-specific reference frameworks.

## 2. Materials and Methods

### 2.1. Study Design and Population

This retrospective, cross-sectional observational study was conducted at a single tertiary referral center. The study protocol was approved by the Non-Interventional Clinical Research Ethics Committee of Gaziantep City Hospital, and the investigation was performed in accordance with the principles of the Declaration of Helsinki (Approval No: 385/026, Date: 21 January 2026). Because of the retrospective nature of the study and the use of anonymized archival data, informed consent was waived by the ethics committee.

Cardiac magnetic resonance (CMR) examinations performed between 1 August 2022 and 15 October 2025 were retrospectively screened through the institutional radiology archive system. Adult patients (≥18 years) who had undergone clinically indicated CMR and whose reports included complete measurements of left ventricular (LV) wall thickness (septal and posterolateral segments) and left ventricular end-diastolic diameter (LVEDD) were eligible for inclusion. CMR examinations were performed for routine clinical indications, including evaluation of nonspecific cardiac symptoms, suspected structural heart disease, arrhythmia assessment, atypical chest pain, or exclusion of cardiomyopathy. Patients with overt structural cardiac abnormalities identified on final CMR evaluation, including significant valvular disease, cardiomyopathy, myocarditis, congenital heart disease, or other major structural pathology, were excluded from the final study cohort.

### 2.2. Eligibility Criteria

Adult patients (≥18 years) who underwent clinically indicated cardiac magnetic resonance (CMR) imaging during the study period were eligible for inclusion. Participants were required to have complete and standardized measurements of LV wall thickness and LVEDD documented in the CMR report. Only examinations with adequate image quality, free from significant motion artifacts or technical errors that could compromise measurement reliability, were included. Additionally, accessible demographic information (age and sex) and complete clinical and imaging data within the institutional archival records were mandatory for study eligibility.

Patients were excluded if they were younger than 18 years, had incomplete or non-standardized LV wall thickness or LVEDD measurements, or if image quality was insufficient for accurate assessment. Individuals with significant valvular heart disease, congenital cardiac anomalies, or pericardial disease that could independently alter LV geometry were excluded to avoid confounding structural effects. Pregnant or lactating women were also excluded. Furthermore, cases with missing, inconsistent, or non-anonymizable identification data were not included in the analysis. Patients with restricted legal status, including prisoners or individuals lacking decision-making capacity, were also excluded from the study.

**Figure 1:**
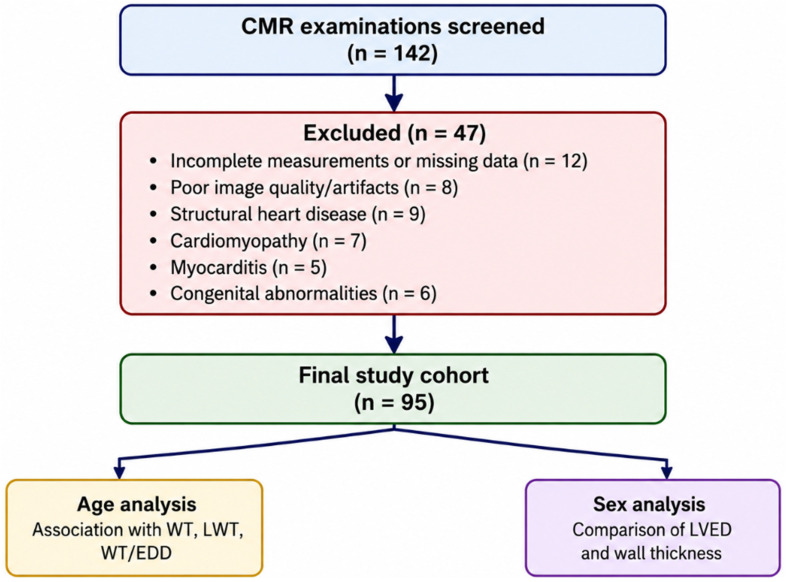
Flowchart of the Study.

Sample size estimation was performed using G*Power version 3.1. Based on an anticipated medium effect size (f = 0.35), α = 0.05, and statistical power of 0.80 for ANOVA comparisons across age groups, the minimum required sample size was calculated as 84 participants (Figure 1).

### 2.3. CMR Acquisition Protocol

All CMR examinations were performed using a 1.5 Tesla scanner (Philips Healthcare, Best, The Netherlands) with a standardized institutional imaging protocol. Cine images were acquired using a steady-state free precession (SSFP) sequence, in accordance with current Society for Cardiovascular Magnetic Resonance (SCMR) recommendations for morphological and functional assessment [11]. Standard long-axis and short-axis views were obtained during breath-hold acquisitions. LV wall thickness measurements were obtained at end-diastole from the interventricular septal wall and the posterolateral LV wall using standardized cine-CMR images according to the institutional imaging protocol. Left ventricular end-diastolic diameter (LVEDD) measurements were obtained from standardized four-chamber cine images at end-diastole according to the institutional imaging protocol. All measurements were retrospectively reviewed and confirmed directly from archived cine-CMR images in addition to routine diagnostic reports. No additional imaging or reprocessing beyond standard clinical practice was performed.

### 2.4. Image Analysis and Measurement Standardization

All CMR measurements were reviewed and confirmed by a single experienced radiologist (≥10 years of experience in cardiovascular imaging), who was blinded to the study hypothesis during measurement validation. Wall thickness values (in millimeters) were recorded for septal and posterolateral segments at end-diastole. The mean wall thickness was calculated when appropriate. LVEDD (mm) was extracted from the standardized report.

To characterize proportional LV geometry, relative wall thickness (RWT) was calculated using the following formula: RWT = (2 × posterior wall thickness)/LV end-diastolic diameter (LVEDD). Concentric remodeling was defined as an RWT value ≥0.42, consistent with commonly used remodeling criteria in cardiovascular imaging literature [26,33]. Because this retrospective study was primarily based on routine report-derived morphologic measurements, systematic left ventricular mass index (LVMI) data were not consistently available for all participants. Therefore, although the classical four-category LV geometry framework (normal geometry, concentric remodeling, concentric hypertrophy, and eccentric hypertrophy) was considered conceptually, the primary geometric analysis in the present study was based on a binary classification approach (normal geometry vs. concentric remodeling) derived from RWT criteria.

**Figure 2:**
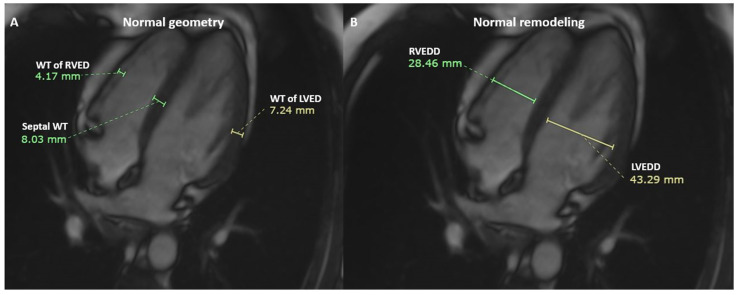
Representative cine-CMR images demonstrating differences in LV geometry and proportional remodeling patterns. (**A**) Representative example of normal LV geometry with lower relative wall thickness. (**B**) Representative example of normal remodeling with increased wall thickness relative to LVEDD. Measurement overlays indicate LV wall thickness and LVEDD parameters used for RWT and WT/LVEDD calculations.

Geometric classification was determined using wall thickness and LVEDD values, and when available, left ventricular mass index (LVMI) was incorporated as a supportive parameter. To enhance measurement reliability, all extracted parameters were double-checked. All measurements were reviewed by a single experienced radiologist using a standardized measurement workflow. Interobserver reproducibility was not assessed because measurements were performed by a single observer. Representative CMR images illustrating LVEDD and wall thickness measurements used for geometric assessment are provided in Figure 2.

### 2.5. Clinical and Demographic Variables

Demographic and clinical data were retrospectively retrieved from the institutional medical archive system. The primary demographic variables included age (years) and sex (male/female). For subgroup analyses, participants were categorized into three predefined age groups: 18–40 years, 41–60 years, and >60 years.

Clinical variables recorded included the presence of hypertension, diabetes mellitus, dyslipidemia, chronic obstructive pulmonary disease, and chronic kidney disease. Several retrospective clinical variables, including BMI, hypertension, diabetes mellitus, smoking status, and medication use, were incompletely available within the institutional archive system and were therefore analyzed descriptively when accessible. In addition, the use of medications known to potentially influence cardiac structure—such as beta-blockers, angiotensin-converting enzyme (ACE) inhibitors, and diuretics—was recorded. Heart rate (beats per minute) measured during CMR acquisition was included as a physiological covariate to account for potential hemodynamic variability affecting chamber dimensions.

### 2.6. Outcome Measures

The primary geometric outcome was binary LV geometric status (normal geometry vs. concentric remodeling) based on RWT classification. Because systematic LVMI data were not available for all participants, the complete four-category LV geometry classification system could not be fully implemented. Secondary outcomes included age-related differences in LV morphological parameters and sex-based variations in LV geometric indices. These analyses aimed to determine whether demographic factors independently influenced structural remodeling patterns after adjustment for relevant clinical covariates.

### 2.7. Statistical Analysis

All statistical analyses were performed using IBM SPSS Statistics version 26.0 (IBM Corp., Armonk, NY, USA). A two-tailed *p*-value <0.05 was considered statistically significant. Continuous variables were expressed as mean ± standard deviation (SD) for normally distributed data and as median (minimum–maximum) for non-normally distributed data. Categorical variables were presented as frequencies (n) and percentages (%). The distribution of continuous variables was assessed using the Shapiro–Wilk test, supported by visual inspection of histograms and Q–Q plots. Homogeneity of variances was evaluated using Levene’s test when appropriate. For sex-based comparisons, normally distributed continuous variables were analyzed using the independent samples *t*-test, whereas the Mann–Whitney U test was applied for non-normally distributed variables. Comparisons across predefined age groups (18–40, 41–60, >60 years) were performed using one-way analysis of variance (ANOVA) when normality and homogeneity assumptions were satisfied. In cases where these assumptions were violated, the Kruskal–Wallis test was used. Post hoc pairwise comparisons were conducted using Tukey’s test following ANOVA or Dunn’s test following Kruskal–Wallis analysis, with appropriate adjustment for multiple comparisons. Categorical variables, including LV geometric pattern distribution, were compared using the chi-square test or Fisher’s exact test when cell counts were small. Associations between age, LV wall thickness, and LVEDD were evaluated using Pearson correlation analysis for normally distributed variables and Spearman rank correlation analysis for non-normally distributed variables. Correlation coefficients (r) were interpreted according to standard effect size conventions.

To identify independent associated factors of LV geometric parameters, multivariable linear regression analyses were performed. LVEDD and the wall thickness-to-end-diastolic diameter ratio (WT/EDD) were analyzed as continuous dependent variables in separate regression models. Age and sex were included as the primary covariates in the final multivariable regression models because these variables were consistently available across the study population. BMI, hypertension, and diabetes mellitus were initially evaluated as candidate covariates; however, these variables contained incomplete data in the retrospective archive-derived dataset and were therefore not retained in the final regression models to avoid a substantial reduction in effective sample size and model instability. Complete-case analysis was applied for all multivariable regression models. Variable selection was based on established clinical relevance and prior evidence linking these factors to myocardial remodeling. A forced-entry (enter) method was applied to ensure simultaneous adjustment for all covariates. For logistic regression analysis, the dependent outcome variable was binary LV geometry classification (normal geometry vs. concentric remodeling). Regression diagnostics were conducted to verify model assumptions. Linearity between associated factors and dependent variables was assessed using partial regression plots. Independence of residuals was evaluated using the Durbin–Watson statistic. Homoscedasticity was examined through residual scatterplots, and normality of residuals was assessed using a histogram and Q–Q plot inspection. Multicollinearity was evaluated using the variance inflation factor (VIF) and tolerance values, with VIF > 5 considered indicative of potential collinearity concerns. The results were reported as unstandardized coefficients (B), standardized beta coefficients (β), 95% confidence intervals (CI), and corresponding *p*-values. Model fit and explanatory capacity were quantified using adjusted R^2^ values. Full multivariable regression outputs and covariate availability are provided in Supplementary Table S1.

## 3. Results

Most participants underwent clinically indicated CMR examinations for exclusion of structural cardiac disease or evaluation of nonspecific cardiovascular symptoms. Major structural cardiac abnormalities were excluded from the final cohort according to predefined eligibility criteria. A total of 95 patients were included in the analysis. The mean age of the study population was 34.94 ± 16.00 years. Of the participants, 57 (60.0%) were male, and 38 (40.0%) were female. No cases of valve pathology, cardiomyopathy, or myocarditis were identified in the study cohort (0%). The mean left ventricular end-diastolic diameter was 41.78 ± 6.72 mm, and the mean right ventricular end-diastolic diameter was 33.20 ± 8.02 mm. The mean left ventricular wall thickness was 8.92 ± 4.20 mm. The average relative wall thickness (RWT) was 0.41 ± 0.23, and the mean WT/LVEDD ratio was 0.22 ± 0.12 (Table 1).

The comparison of LV morphological parameters according to sex is shown in Table 2. Male participants demonstrated significantly greater LV end-diastolic diameter compared to females (43.12 ± 6.83 mm vs. 39.76 ± 6.11 mm, *p* = 0.014). Interventricular septal thickness was also significantly higher in males (10.51 ± 4.91 mm) than in females (8.32 ± 3.80 mm, *p* = 0.016). Similarly, posterior wall thickness was greater in males (8.86 ± 4.41 mm vs. 7.21 ± 3.05 mm, *p* = 0.034). Mean LV wall thickness was also significantly greater in males (9.68 ± 4.58 mm vs. 7.76 ± 3.26 mm, *p* = 0.019). No statistically significant differences were observed between sexes in relative wall thickness (0.43 ± 0.26 vs. 0.37 ± 0.17, *p* = 0.212), or WT/LVEDD ratio (0.23 ± 0.13 vs. 0.20 ± 0.09, *p* = 0.144) (Table 2).

The LV morphological parameters according to age groups are shown in Table 3. When stratified according to age groups (18–40 years, 41–60 years, and >60 years), interventricular septal thickness differed significantly among groups (*p* = 0.049). Mean septal thickness progressively increased from 8.94 ± 4.38 mm in the 18–40 group to 11.06 ± 5.13 mm in the 41–60 group and 12.10 ± 4.33 mm in participants older than 60 years. Posterior wall thickness showed an increasing trend across age categories (7.64 ± 3.82 mm, 9.56 ± 4.90 mm, and 9.90 ± 2.64 mm, respectively), although this difference did not reach statistical significance (*p* = 0.079). Similarly, mean LV wall thickness increased across age groups (8.29 ± 3.99 mm, 10.31 ± 4.92 mm, and 11.00 ± 3.40 mm), with borderline statistical significance (*p* = 0.054). Relative wall thickness (RWT) and WT/LVEDD ratio also demonstrated increasing mean values with advancing age (RWT: 0.38 ± 0.21, 0.46 ± 0.33, 0.49 ± 0.16; WT/LVEDD: 0.21 ± 0.11, 0.25 ± 0.16, 0.27 ± 0.10), without statistically significant differences between groups (*p* = 0.192 and *p* = 0.148, respectively). No significant difference was observed in LV end-diastolic diameter across age groups (41.16 ± 6.42 mm, 44.75 ± 8.04 mm, and 41.30 ± 5.85 mm; *p* = 0.153) (Table 3).

**Figure 3:**
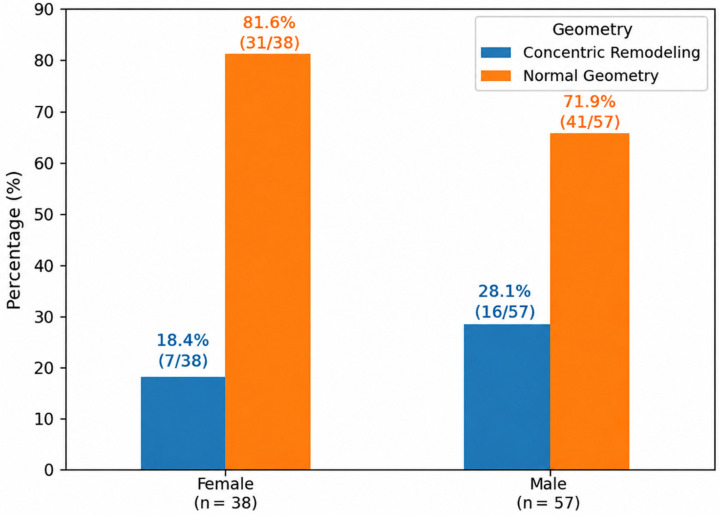
Distribution of binary LV geometry classification (normal geometry vs. concentric remodeling) according to sex. Absolute counts and percentages are shown for each category.

The distribution of binary LV geometric classification (normal geometry vs. concentric remodeling) did not significantly differ between sexes. Concentric remodeling was observed in 16 of 57 male participants (28.1%) and 7 of 38 female participants (18.4%), whereas normal geometry was present in 41 of 57 males (71.9%) and 31 of 38 females (81.6%) (*p* = 0.311) (Figure 3).

**Figure 4:**
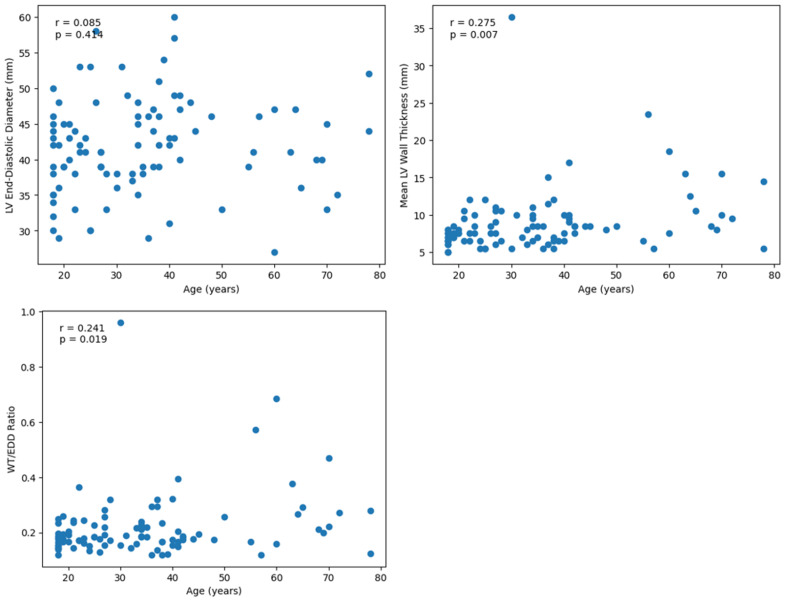
Correlation Between Age and LV Morphological Parameters.

Age showed a weak and non-significant correlation with left ventricular end-diastolic diameter (r = 0.085, *p* = 0.414). In contrast, age demonstrated a statistically significant positive correlation with mean left ventricular wall thickness (r = 0.275, *p* = 0.007). Similarly, a significant positive correlation was observed between age and the wall thickness-to-end-diastolic diameter ratio (WT/LVEDD) (r = 0.241, *p* = 0.019) (Figure 4).

Final multivariable regression models were constructed using age and sex as covariates based on complete-case availability. Multivariable linear regression analysis adjusted for age and sex is shown in Table 4. In multivariable linear regression analysis, male sex was independently associated with larger LV end-diastolic diameter (B = 3.345, 95% CI: 0.614–6.076, *p* = 0.017), whereas age was not a significant associated factor (*p* = 0.420). In the model evaluating the WT/LVEDD ratio, age remained an independent associated factor (B = 0.0018, 95% CI: 0.0003–0.0033, *p* = 0.019), while sex was not significantly associated (*p* = 0.179) (Table 4).

In logistic regression analysis evaluating associated factors of concentric remodeling, age was independently associated with increased odds of concentric geometry (OR = 1.041 per year increase, 95% CI: 1.011–1.072, *p* = 0.006). Male sex was not an independent associated factor (*p* = 0.225) (Table 5).

Detailed regression outputs and covariate availability are presented in Supplementary Table S1.

## 4. Discussion

In the present study, concentric remodeling was operationalized using an RWT threshold of ≥0.42, calculated as (2 × posterior wall thickness)/LV end-diastolic diameter. Because systematic left ventricular mass index (LVMI) measurements were not available for all participants, the analysis was restricted to a binary geometric classification framework (normal geometry vs. concentric remodeling) rather than the complete four-category LV geometry model. This pragmatic approach was considered appropriate for a retrospective dataset derived from routine clinical CMR reports. Within this retrospective clinical cohort, sex appeared to be more strongly associated with absolute cardiac dimensions, whereas advancing age showed associations with relative wall–cavity geometric indices reflecting concentric remodeling tendencies.

Riffel et al. showed robust age- and sex-related differences in CMR-derived cardiac morphology, with men showing larger ventricular dimensions and volumes compared with women across age strata [26]. Our results are concordant: male participants demonstrated larger absolute LV dimensions and wall thickness measurements; however, these findings should be interpreted cautiously because body surface area indexing was not available in the present dataset. Similar sex-associated differences have also been documented in large CMR reference datasets and consensus compilations, where males consistently exhibit larger cavity dimensions and higher absolute mass-related measures [12,33]. The alignment of our findings with these reference studies suggests that routine-report morphometrics can capture expected biological variation, even without advanced post-processing.

An additional observation in our dataset was the lack of a statistically significant sex difference in relative indices (RWT and WT/LVEDD). This is consistent with a scenario in which both wall thickness and cavity size scale upward in men, producing higher absolute values but similar proportional geometry. Gregor et al., using CMR-based quantification approaches, emphasized that sex effects are pronounced for absolute structural parameters, while relationships between certain normalized measures may attenuate within age strata [34]. Taken together, these results reinforce the importance of reporting both absolute (mm) and proportional (ratio-based) indices when interpreting remodeling signals across sexes. Future studies incorporating systematic BSA-indexed morphologic analyses may help better distinguish biological sex-related variation from differences attributable to overall body size.

Age exhibited a significant positive association with interventricular septal thickness and showed positive correlations with mean wall thickness and WT/LVEDD ratio. In multivariable analysis, age remained an independent associated factor of WT/LVEDD, and in logistic modeling, age independently increased the odds of concentric remodeling. These findings are directionally consistent with previously reported observations related to cardiac aging; however, they should be interpreted cautiously given the relatively young study population and limited number of elderly participants. These findings should therefore be interpreted as exploratory observations within this retrospective clinical cohort rather than definitive evidence of late-life remodeling patterns.

Peverill et al. summarized evidence that healthy aging is typically associated with reductions in LV volumes and changes in geometry, with complex relationships among LV size, filling pressures, and indices of pump performance [25]. In our cohort, LVEDD did not significantly vary across age groups and did not correlate significantly with age. This apparent discrepancy from studies showing age-related decreases in LV size may be explained by the relatively young mean age of our sample and the modest number of participants >60 years, which may limit sensitivity to detect late-life reductions in cavity dimensions. Moreover, our measurements were derived from routine reports rather than fully indexed volumetric parameters (e.g., LVEDD indexed to BSA), which may attenuate detection of subtler age-related size trends described in population cohorts.

We operationalized concentric remodeling using an RWT threshold (≥0.42), a commonly used criterion in remodeling literature and epidemiologic work [35]. While RWT is traditionally computed as 2 × posterior wall thickness divided by LV diastolic diameter, it remains a practical, interpretable marker of concentricity that can be derived from routine measurements. The clinical relevance of RWT is supported by outcome-oriented studies in selected cardiovascular populations, in which RWT has been linked to arrhythmic risk and remodeling trajectories, underscoring that wall cavity proportion captures meaningful biology beyond absolute thickness alone [36].

Notably, the distribution of remodeling patterns did not significantly differ by sex in our cohort (chi-square *p* = 0.311), whereas age showed a robust signal in the regression framework. This pattern is internally consistent: sex was primarily associated with absolute morphology (LVEDD and wall thickness), whereas advancing age was associated with proportional geometric indices within this specific retrospective cohort, including WT/LVEDD and RWT-defined remodeling. In practical terms, this suggests that clinicians interpreting routine CMR report metrics may expect male patients to present with larger absolute dimensions without necessarily implying a higher burden of concentric remodeling when proportional indices remain within expected ranges.

We specifically tested whether the association between age and WT/LVEDD differed by sex (Age × Sex interaction) and found no significant interaction. This implies that the slope of age-related change in WT/LVEDD is broadly similar in males and females in our dataset. Riffel et al. highlighted that both sexes demonstrate age-dependent shifts in CMR morphology, although baseline levels differ [26]. Our findings extend this by suggesting that, at least for the proportional measure WT/LVEDD within our cohort, aging effects appear directionally consistent across sexes.

From a clinical standpoint, our results support the feasibility of extracting remodeling-relevant information from routine CMR reports without requiring advanced segmentation. Standardized acquisition and reporting frameworks promoted by SCMR facilitate comparability and reproducibility, making report-derived morphometrics suitable for retrospective research and potentially for pragmatic clinical decision support [11]. In settings where comprehensive volumetric/mass quantification is not systematically performed, ratio-based indices such as WT/LVEDD or RWT may provide a low-burden method to flag early concentric remodeling tendencies that track with aging.

Current ESC/EACVI and SCMR-oriented cardiac imaging recommendations emphasize the importance of standardized structural assessment and reproducible morphologic reporting in the evaluation of ventricular remodeling and cardiovascular risk stratification. Although comprehensive volumetric and mass-indexed analyses remain the preferred reference standard, routine report-derived morphologic indices such as LV wall thickness, LVEDD, WT/LVEDD ratio, and RWT may provide practical complementary information in everyday clinical CMR interpretation, particularly in retrospective datasets or resource-limited settings where advanced post-processing is not systematically available.

Unlike previously published CMR reference studies that primarily focused on establishing age- and sex-specific normative ranges for ventricular volumes, mass, and functional parameters in large population cohorts [12,26,33], our study specifically evaluated routinely reported linear morphologic indices (wall thickness and end-diastolic diameter) and their proportional relationship (WT/LVEDD) as practical markers of geometric remodeling. While prior investigations such as Riffel et al. and Petersen et al. emphasized volumetric and mass-indexed measurements derived from dedicated research protocols [12,26], our analysis demonstrates that standard report-based parameters obtained in daily clinical practice can capture biologically meaningful age-dependent concentric remodeling signals independent of sex. Moreover, by integrating logistic regression and interaction analysis, we delineated a clear dissociation between sex-driven chamber enlargement and age-driven proportional geometric adaptation—an aspect not explicitly modeled in earlier CMR reference studies. Thus, rather than redefining normative values, our work extends existing literature by translating structural remodeling assessment into a pragmatic, report-based framework applicable to real-world clinical CMR data.

This study has several strengths. First, it leverages routine, clinically acquired CMR data using standardized protocols, enhancing translational relevance. Second, the measurements were validated within a consistent workflow, and the analytic approach integrated complementary methods (group comparisons, correlation, linear regression, logistic regression, and interaction testing) to triangulate findings.

Limitations should be considered. The retrospective, single-center design may limit generalizability and introduce potential selection bias. Referral indications and retrospective clinical selection may also have influenced baseline cardiac morphology, thereby limiting the external generalizability of the findings. In addition, the relatively small number of participants in the older age strata, particularly those older than 60 years, may have reduced statistical power to detect age-related differences in LVEDD and limited the generalizability of the observed age-related associations, especially in subgroup analyses. Because the original sample size calculation was based on overall cohort comparisons rather than elderly subgroup-specific analyses, the study may have been underpowered for detecting subtle age-related geometric differences in participants older than 60 years. Because LV mass and LVMI measurements were not systematically available in all patients, the classical four-category LV geometry classification system could not be fully implemented. Therefore, the present study relied on a binary RWT-based remodeling classification approach. Formal interobserver and intraobserver reproducibility analyses were not performed, which may limit assessment of measurement variability. In addition, body surface area (BSA) indexing was not systematically available and therefore indexed morphologic analyses could not be performed. Consequently, the observed sex-related differences in LV dimensions may partly reflect differences in overall body size rather than isolated biological sex effects. Furthermore, several retrospective clinical variables such as BMI, hypertension, and diabetes mellitus were incompletely available in the archive-derived dataset, limiting the feasibility of more comprehensively adjusted multivariable analyses.

Future prospective, multicenter work incorporating standardized volumetric and mass quantification (including LVMI and indexed volumes) could validate and extend these findings, enable full four-category geometry classification, and link remodeling patterns to clinical outcomes. The integration of age- and sex-adjusted reference frameworks from large population cohorts may further improve interpretability and facilitate development of locally applicable normative ranges, particularly in diverse populations.

## 5. Conclusions

In conclusion, within this retrospective clinical cohort, advancing age showed associations with proportional changes in left ventricular geometry, reflected by higher WT/LVEDD ratios and a greater prevalence of RWT-defined concentric remodeling. Male participants tended to demonstrate larger absolute chamber dimensions; however, the contribution of overall body size could not be fully evaluated because indexed analyses were unavailable. These findings suggest that routine CMR report-derived linear measurements may provide practical insight into LV geometric variation in daily clinical practice. Nevertheless, given the retrospective design, the limited number of elderly participants, and the absence of comprehensive indexed volumetric analyses, the findings should be interpreted cautiously and require validation in larger prospective studies.

## Figures and Tables

**Table 1:** Baseline Demographic, Clinical, and CMR Characteristics of the Study Population.

Variable	Patients (n = 95)
Age (years)	34.94 ± 16.00
Male sex	57 (60.0%)
Female sex	38 (40.0%)
Clinical Variables	
Hypertension	12 (12.6%)
Diabetes mellitus	6 (6.3%)
Dyslipidemia	10 (10.5%)
Current smoking	18 (18.9%)
COPD	3 (3.2%)
Chronic kidney disease	2 (2.1%)
BMI available	41 (43.2%)
Heart rate during CMR (beats/min)	76.2 ± 11.4
Beta-blocker use	9 (9.5%)
ACE inhibitor/ARB use	11 (11.6%)
Diuretic use	4 (4.2%)
Significant valvular disease (Cardiomyopathy, Myocarditis, Congenital heart disease.)	0 (0.0%)
CMR Morphologic Parameters	
LVEDD (mm)	41.78 ± 6.72
Mean LV wall thickness (mm)	8.92 ± 4.20
Relative wall thickness (RWT)	0.41 ± 0.23
WT/LVEDD ratio	0.22 ± 0.12

ACE, angiotensin-converting enzyme; ARB, angiotensin receptor blocker; BMI, body mass index; CMR, cardiac magnetic resonance; COPD, chronic obstructive pulmonary disease; LV, left ventricular; LVEDD, left ventricular end-diastolic diameter; RWT, relative wall thickness; WT, wall thickness.

**Table 2:** Comparison of LV Morphological Parameters According to Sex.

Parameter	Male (n = 57)	Female (n = 38)	***p***-Value
LV End-Diastolic Diameter (mm)	43.12 ± 6.83	39.76 ± 6.11	0.014
Interventricular Septal Thickness (mm)	10.51 ± 4.91	8.32 ± 3.80	0.016
Posterior Wall Thickness (mm)	8.86 ± 4.41	7.21 ± 3.05	0.034
Mean LV Wall Thickness (mm)	9.68 ± 4.58	7.76 ± 3.26	0.019
Relative Wall Thickness (RWT)	0.43 ± 0.26	0.37 ± 0.17	0.212
WT/LVEDD Ratio	0.23 ± 0.13	0.20 ± 0.09	0.144

**Table 3:** LV Morphological Parameters According to Age Groups.

Parameter	18–40 (n = 69)	41–60 (n = 16)	>60 (n = 10)	***p***-Value
LV End-Diastolic Diameter (mm)	41.16 ± 6.42	44.75 ± 8.04	41.30 ± 5.85	0.153
Interventricular Septal Thickness (mm)	8.94 ± 4.38	11.06 ± 5.13	12.10 ± 4.33	0.049
Posterior Wall Thickness (mm)	7.64 ± 3.82	9.56 ± 4.90	9.90 ± 2.64	0.079
Mean LV Wall Thickness (mm)	8.29 ± 3.99	10.31 ± 4.92	11.00 ± 3.40	0.054
Relative Wall Thickness (RWT)	0.38 ± 0.21	0.46 ± 0.33	0.49 ± 0.16	0.192
WT/LVEDD Ratio	0.21 ± 0.11	0.25 ± 0.16	0.27 ± 0.10	0.148

**Table 4:** Multivariable Linear Regression Analysis Adjusted for Age and Sex.

Model 1—Dependent Variable: LV End-Diastolic Diameter (LVEDD)			
Associated Factor	B	95% CI	***p***-Value
Age	0.034	−0.050 to 0.118	0.420
Male sex	3.345	0.614 to 6.076	0.017
R^2^ = 0.067, Adjusted R^2^ = 0.047, Overall model *p* = 0.041			
**Model 2**—Dependent Variable: WT/LVEDD Ratio			
**Associated factor**	**B**	**95% CI**	***p*** **-value**
Age	0.0018	0.0003 to 0.0033	0.019
Male sex	0.032	−0.015 to 0.080	0.179
R^2^ = 0.076, Adjusted R^2^ = 0.056, Overall model *p* = 0.026			

**Table 5:** Binary Logistic Regression Analysis Adjusted for Age and Sex.

Associated Factor	OR	95% CI	***p***-Value
Age	1.041	1.011–1.072	0.006
Male sex	1.902	0.673–5.375	0.225

## Data Availability

The original contributions presented in this study are included in the article/Supplementary Materials.

## Supplementary Materials

The following supporting information can be downloaded at: https://www.mdpi.com/article/10.3390/tomography12060090/s1. Table S1: Full Multivariable Regression Outputs and Covariate Availability.

## Abbreviations

BMI	Body Mass Index
CI	Confidence Interval
CMR	Cardiac Magnetic Resonance
EDD	End-Diastolic Diameter
IVSd	Interventricular Septal Thickness in Diastole
LV	Left Ventricle/Left Ventricular
LVEDD	Left Ventricular End-Diastolic Diameter
OR	Odds Ratio
PWTd	Posterior Wall Thickness in Diastole
RWT	Relative Wall Thickness
WT	Wall Thickness
WT/LVEDD	Wall Thickness-to-LV End-Diastolic Diameter Ratio
