# Supplementary material for: Age-Related Concentric Remodeling and Sex-Dependent Dimensional Variation in Left Ventricular Geometry: A Cardiac Magnetic Resonance Study
Source: Tomography. 2026 Jun 22;12(6):90. doi: 10.3390/tomography12060090 (PMC13306415; doi:10.3390/tomography12060090)
Supplement: Supplementary file 1 [file tomography-12-00090-s001.zip › tomography-4238129-supplementary.pdf]

Supplementary Table S1. Full Multivariable Regression Outputs and Covariate Availability.

Model	Variable	Effect Estimate	95% CI	p-value
Model 1: Multivariable Linear Regression for LVEDD	Age (years)	B = 0.034	- 0.050 – 0.118	0.420
	Male sex	B = 3.345	0.614 – 6.076	0.017
	BMI	NA*	NA	NA
	Hypertension	NA*	NA	NA
	Diabetes mellitus	NA*	NA	NA
	Adjusted R^2	0.047		
	Final model sample size	n = 95		
**Model 2: Multivariable Linear Regression for WT/EDD Ratio**	Age (years)	B = 0.0018	0.0003 – 0.0033	0.019
	Male sex	B = 0.032	-0.015 – 0.080	0.179
	BMI	NA*	NA	NA
	Hypertension	NA*	NA	NA
	Diabetes mellitus	NA*	NA	NA
	Adjusted R^2	0.056		
	Final model sample size	n = 95		
**Model 3: Binary Logistic Regression for Concentric Remodeling**	Age (years)	OR = 1.041	1.011 – 1.072	0.006
	Male sex	OR = 1.902	0.673 – 5.375	0.225
	BMI	NA*	NA	NA
	Hypertension	NA*	NA	NA
	Diabetes mellitus	NA*	NA	NA
	Final model sample size	n = 95		

Abbreviations: BMI, body mass index; CI, confidence interval; LVEDD, left ventricular end-diastolic diameter; OR, odds ratio; WT/EDD, wall thickness-to-end-diastolic diameter ratio.

* BMI, hypertension, and diabetes mellitus were initially evaluated as candidate covariates; however, these variables were incompletely available in the retrospective archive-derived dataset and were therefore not retained in the final multivariable models to avoid substantial reduction in effective sample size and model instability.
